# Lumican modulates adipocyte function in obesity-associated type 2 diabetes

**DOI:** 10.1080/21623945.2022.2154112

**Published:** 2022-12-06

**Authors:** Strieder-Barboza, Carmen G. Flesher, Lynn M. Geletka, Tad Eichler, Olukemi Akinleye, Alexander Ky, Anne P. Ehlers, Carey N. Lumeng, Robert W. O’Rourke

**Affiliations:** a Department of Surgery , University of Michigan Medical School, MI, USA; b Department of Veterinary Sciences, Texas Tech University, Lubbock , TX, USA; c School of Veterinary Medicine, Texas Tech University, Amarillo, TX, USA; d Department of Pediatrics and Communicable Diseases, University of Michigan, Ann Arbor, MI, USA; e Department of Surgery, Veterans Affairs Ann Arbor Healthcare System, Ann Arbor, MI, USA; f Graduate Program in Immunology, University of Michigan Medical School, Ann Arbor, MI ,USA; g Graduate Program in Cellular and Molecular Biology, University of Michigan Medical School, Ann Arbor, MI, USA

**Keywords:** Lumican, adipose tissue, diabetes, insulin resistance, ERK

## Abstract

Obesity-associated type 2 diabetes (DM) leads to adipose tissue dysfunction. Lumican is a proteoglycan implicated in obesity, insulin resistance (IR), and adipocyte dysfunction. Using human visceral adipose tissue (VAT) from subjects with and without DM, we studied lumican effects on adipocyte function. Lumican was increased in VAT and adipocytes in DM. Lumican knockdown in adipocytes decreased lipolysis and improved adipogenesis and insulin sensitivity in VAT adipocytes in DM, while treatment with human recombinant lumican increased lipolysis and impaired insulin-sensitivity in an ERK-dependent manner. We demonstrate that lumican impairs adipocyte metabolism, partially via ERK signalling, and is a potential target for developing adipose tissue-targeted therapeutics in DM.

## Introduction

Obesity-associated type 2 diabetes (DM) is a major cause of global morbidity and mortality [1]. Adipose tissue is a promising target for novel DM therapies[2], as DM pathogenesis stems in part from unhealthy expansion of adipose tissue in obesity, leading to adipocyte insulin resistance (IR). Aberrant extracellular matrix proteins deposition (ECM) is a particularly important contributor to adipose tissue dysfunction in obesity. Multiple reports establish a link between aberrant adipose tissue ECM and obesity and metabolic disease, while our group has demonstrated that the adipose tissue ECM regulates adipocyte metabolism in a DM- and depot-specific manner [3–8]. Nonetheless, the specific mechanisms involved in ECM regulation of adipose tissue metabolism in DM remain unclear.

Lumican (encoded by *LUM* gene) is a ubiquitous class II small leucine-rich proteoglycan (SLRP) that regulates ECM composition and collagen fibrillogenesis [9]. Published data regarding the role of lumican in AT metabolism are sparse. *LUM* expression is elevated in visceral adipose tissue (VAT) relative to subcutaneous adipose tissue in mice and humans [10], and in VAT in humans with gestational DM [11] and in the kidneys of humans with diabetic nephropathy [12]. A recent study demonstrated an increase in pericellular LUM in AT of insulin-resistant compared to glucose-tolerant obese humans, along with an anti-adipogenic role for LUM in 3T3-L1 cells [9]. These observations suggest a positive association of lumican with metabolic dysfunction. Through its role in regulating collagen fibril assembly, lumican promotes epithelial wound healing by enhancing α2β1 integrin-mediated fibroblast contractibility [13], recruiting neutrophils to sites of inflammation [14], and inducing cell migration by activating ERK signalling in numerous cell types [15,16]. Lumican also exerts direct effects on cell function in a number of models. Lumican promotes cell proliferation in human trophoblast cells [17], inhibits bone formation and osteoclast function via Akt and ERK signalling [18,19] and exerts both pro- and anti-proliferative effects on a number of cancers [20]. However, the role of lumican in regulating human adipocyte metabolism in human DM has not been studied.

The goal of this manuscript is to define the functional role of lumican in adipocytes in the context of metabolic disease. We hypothesized that lumican expression is increased in VAT in DM and exerts detrimental effects on adipocyte metabolism. We revealed that lumican is increased in VAT and adipocytes from human subjects with DM and impairs adipocyte metabolism, and we implicate ERK signalling in these effects. Our results suggest lumican as a link between the ECM and adipocyte dysfunction and a target for manipulating adipocyte function in DM.

## Material and methods

### Human subjects

Informed consent was obtained, and human subjects research was performed with approval from Institutional Review Boards at the University of Michigan and Veterans Affairs Ann Arbor Healthcare System under guidelines consistent with the 1964 Declaration of Helsinki and the 1974 Belmont Report. Peripheral blood, VAT from the greater omentum was collected from subjects with morbid obesity (BMI ≥ 35) during bariatric surgery at the beginning of the operation. Subjects with DM were defined by clinical diagnosis requiring medication and hemoglobinA1c (HbA1c)≥6.5%. Non-diabetic (NDM) subjects were defined by no clinical history of diabetes and HbA1c < 5.7% per American Diabetes Association criteria. Clinical laboratory values were measured by the clinical laboratories at the University of Michigan and Ann Arbor Veterans Affairs Hospitals (Supplementary Table 1).

### Adipose tissue imaging

VAT was immunostained and imaged for the expression of lumican using a Nikon Eclipse Ti inverted point-scanning confocal microscope (Nikon, Tokyo, Japan) equipped with two standard PMT detectors. VAT fixation, blocking, and permeabilization were performed as described by our group [21], then incubated with lumican primary antibody (Thermo Fisher Scientific Inc., Waltham, MA, USA; # PA5-14,571) at 1:100 dilution for 1 h, RT in blocking buffer (5% BSA in 1X PBS) followed by incubation with secondary antibody (Alexa 568; Thermo Fisher Scientific Inc., Waltham, MA, USA) at 1:250 dilution for 1 h at RT. Samples were then stained with HCS LipidTOX™ Green Neutral Lipid Stain (1:200; Thermo Fisher Scientific Inc., Waltham, MA, USA; # H34475) for 30 min at RT. Immunostaining was followed by serial tissue dehydration with methanol/H2O and overnight clearing in dibenzyl ether (DBE; Sigma-Aldrich, St. Louis, MO, USA). Samples were stored at RT in the dark until imaging.

### Adipocyte culture

Preadipocytes were isolated from the stromal vascular fraction (SVF) of human VAT and digested with Type II collagenase (2 mg/mL in PBS/2% BSA, Life Technologies Inc., Carlsbad, CA, USA), as described [22,23]. SVF was plated and expanded in a basal medium (Dulbecco’s Modified Eagle Medium/Nutrient Mixture F-12; #11330032) supplemented with 15% foetal bovine serum (#26140) and 1% Antibiotic-Antimycotic solution (#15240062), all from Thermo Fisher Scientific Inc. (Waltham, MA, USA). Confluent preadipocytes were induced to differentiate into adipocytes during 14 d of culture in differentiation medium consisting of basal medium supplemented with the following reagents (Sigma-Aldrich, Inc., St. Louis, MO, USA): 10 mg/L transferrin (#10652202001), 33 µM biotin (#B0301), 0.5 µM human insulin (#91077 C), 17 µM pantothenate (#C8731), 100 nM dexamethasone (#D9184), 2 nM 3,3’,5-Triiodo-L-thyronine sodium salt (#T6397), 1 µM ciglitazone (# 230950), 540 µM Isobutyl-1-methylxanthine (#I7018). During gain of function experiments, adipocytes were cultured with human recombinant lumican for 24 h prior to metabolic assays (Prospect Protein Specialists, Israel; # PRO-1821, 2 ug/mL working concentration). Lumican dose was based on previous studies [24,25] and a dose–response assay performed by our lab (Supplementary Figure S1). Adipocytes were treated with 10 µM of U0126 (EMD Millipore/Sigma-Aldrich, St. Louis, MO, USA, #19-147; 10 uM for 24 h, dose and time-course based on previously published literature [15]) to inhibit MEK-ERK signalling prior to metabolic assays.

### Adipocyte LUM gene knockdown

*LUM* knockdown (*LUM KD*) was achieved using RNAiMax Lipofectamine transfection reagent (Invitrogen/Thermo Fisher Scientific Inc., Waltham, MA, USA; #13778075) and siRNA for *LUM* (Sigma-Aldrich, St. Louis, MO, USA; #Hs01_00221303; (5’-3’) GCAACAUCCCUGAUGAGUA [dT][dT]). AnsiRNA, #1 Cyanine 5 (Sigma-Aldrich, St. Louis, MO, USA; #SIC005) and BLOCK-iT Alexa Fluor Red Fluorescent Control (Invitrogen/Thermo Fisher Scientific Inc., Waltham, MA, USA; #14750-100) were used as negative and positive controls, respectively. Lipofectamine (3 mL/well) and siRNA (10 nM/well) were complexed for 15 min at 25°C in antibiotic/antimycotic-free basal medium. The lipofectamine-siRNA complex was then added to cells in the adipocyte differentiation medium and cells were transfected for 48 h at 37°C. *LUM KD* was performed on d 1, 7 or 14 of differentiation *in vitro*, as described in the results section.

### Adipogenesis assays

Adipogenesis was evaluated by (1) Oil red O (ORO) staining [26] in 4% formaldehyde-fixed adipocytes using 0.2% ORO solution (Sigma-Aldrich, St. Louis, MO, USA; #O0625). Absorption of the eluate was measured photometrically at 510 nm. (2) AdipoRed staining (Lonza Inc., Basel, Switzerland; #PT-7009) was performed as instructed by the manufacturer and standardized by total protein/well measured by Pierce BCA assay (Thermo Fisher Scientific Inc., Waltham, MA, USA; #23225). (3) HCS LipidTOX™ staining (Invitrogen/Thermo Fisher Scientific Inc., Waltham, MA, USA; #H344-75/76) was performed in 4% formaldehyde-fixed adipocytes (20 min, RT) using a 1:200 stain:1X PBS dilution (30 min, RT). Cells’ nuclei were then DAPI stained (1 ug/mL; 5 min, RT) and adipocytes imaged with a 20X objective using a Nikon Eclipse Ti inverted point-scanning confocal microscope (Nikon, Tokyo, Japan) equipped with two standard PMTs. Three to four images were obtained from different fields of each well. Total lipid accumulation was assessed by ImageJ, and total relative fluorescence units (RFU) per well were recorded and averaged for each subject. RFU was standardized by cell number as quantified by the number of DAPI-stained nuclei per image.

### Lipolysis assay

*A*dipocytes were insulin and serum starved for 4 h at 37°C with warm 0.1% BSA phenol red free basal medium. Lipolysis was induced with 1 uM isoproterenol (Sigma-Aldrich, St. Louis, MO, USA; #I6504) for 2 h at 37°C in 3% BSA KRBH (135 mM NaCl, 5 mM KCl, 1 mM MgSO4, 0.4 mM K2HPO4, 1 mM CaCl2, 5.5 mM Glucose, 20 mM HEPES; pH 7.4) and assessed by glycerol concentrations in supernatants (Glycerol Assay Kit, Sigma-Aldrich, St. Louis, MO, USA; #MAK117), calibrated by cell DNA content (CyQUANT, Thermo Fisher Scientific Inc., Waltham, MA, USA; #C7026). Glycerol concentrations in non-treated wells were considered as basal values.

### Glucose uptake assay

Adipocytes were cultured in insulin and serum-free basal medium at 37°C for 12 h and then in 1% BSA PBS for 2 h, then washed in PBS, and incubated with ±200 nM human insulin in PBS at 37°C for 40 min. Next, the cells were incubated with 0.1 mM 2-deoxy glucose, 2μCi/mL deoxy-D-glucose-2-[1,2-[3]H(N)] (PerkinElmer Inc., Waltham, MA, USA) at 37°C for 40 min, washed with PBS, and 420 mL 1% SDS solution added for inducing cells lysis. Ten microlitres of cell lysate was used for Bradford protein assay (Bio-Rad Laboratories, Hercules, CA, USA) for data calibration. Overall, 400 mL of lysate was transferred to 2 mL scintillation fluid and counts per minute (cpm) were measured on a scintillation counter.

### *Quantitative PCR (qPCR*)

Adipose tissue samples were lysed in Trizol and RNA extracted with RNAeasy Fibrous Tissue MiniKit (Qiagen Inc., Hilden, Germany; #74704). RNA was isolated with the RNeasy Mini Kit (Qiagen Inc., Hilden, Germany; #74104). Equal amounts of input RNA were reverse-transcribed using the Applied Biosystems High Capacity cDNA Archive Kit (Applied Biosystems, Inc., Foster City, CA, USA). qPCR was conducted with TaqMan primers and reagents (Life Technologies Inc., Carlsbad, CA, USA). Data are presented as fold changes calculated from least squares mean differences according to the 2^−ΔΔCt^ method [27] and normalized to *B2M*, or the average expression of *B2M* and *ACTB* housekeeping gene controls. Samples used as calibrators are described in each figure.

### Western blotting

Protein from VAT and cultured adipocytes was extracted using an ice-cold RIPA buffer (Thermo Fisher Scientific Inc., Waltham, MA, USA; #89900) containing protease and phosphatase inhibitors. Protein content was quantified using the Pierce BCA Protein Assay Kit (Thermo Fisher Scientific Inc., Waltham, MA, USA). Samples were added to a reducing buffer containing 10 mmol/L of dithiothreitol and 5% β-mercaptoethanol and denatured in boiling water for 4 min. Equal amounts of protein per treatment were separated by electrophoresis on 4% to 20% SDS-PAGE gels (Bio-Rad Laboratories, Hercules, CA, USA; # 456–1094) and transferred to a low fluorescence PVDF membrane (Bio-Rad Laboratories, Hercules, CA, USA, #1620260). Membranes were blocked for 1 h at RT in Tris-buffered saline (TBS) blocking buffer [5% fatty acid free BSA in TBS, 0.5% Tween-20 (TBST)] and incubated with primary antibodies (1:1,000) for lumican (Thermo Fisher Scientific Inc., Waltham, MA, USA; # PA5-14,571), and the following antibodies from Cell Signalling Technology (Danvers, MA, USA): pAKT [Phospho-Akt (Ser473) (D9E) XP®, #4060], AKT [Akt (pan) (11E7), #4685] in TBST-5% BSA for 16 h at 8°C. β-actin (1:20,000; Invitrogen/Thermo Fisher Scientific Inc., Waltham, MA, USA; #MA5-15,739) served as loading control. Blots were washed three times and Alexa 647 or Alexa 488 secondary antibodies (1:20.000) (Thermo Fisher Scientific Inc., Waltham, MA, USA) applied 1 h at RT in a blocking buffer. Fluorescent detection of bands was performed by Azure c600 (Azure Biosystems, Inc., Dublin, CA, USA) using greyscale imaging. Densitometry was performed using AzureSpot Analysis normalizing data between samples to match actin densitometry signals.

### ELISA

We measured lumican concentrations in serum and VAT lysates from DM and NDM subjects, as well as supernatants from *in vitro* cultured preadipocytes and adipocytes using a human lumican ELISA kit (Sigma-Aldrich, St. Louis, MO, USA; #RAB1571) following instructions provided by the manufacturer. Serum samples were previously diluted at 1:100 with dilution buffer provided in the kit. VAT lysates were generated as described for Western Blotting analysis. Cell culture supernatants from preadipocytes were collected at confluence, while supernatants from adipocytes were collected at the last day of differentiation. Negative control samples included preadipocyte and adipocyte differentiation mediums, FBS, and 1X PBS. Lumican concentrations were not detectable in any of these controls. Lumican concentrations were calibrated by the total protein concentrations of each sample.

### Statistical analyses

GraphPad Prism 9 was used to perform statistical analysis. Data normality was analysed by Shapiro–Wilk test assuming Gaussian distribution. Comparisons between DM and NDM were performed using unpaired two-tailed t-tests. Experiments with more than one treatment were analysed using one-way ANOVA followed by multiple comparison analysis (Tukey or Bonferroni). Associations between blood markers and serum concentrations of lumican were performed using Pearson correlation and linear regression analyses. Analysis of preadipocyte proliferation on different days was performed using repeated measures ANOVA followed by Tukey multiple comparison test. Effects were considered significant when P < 0.05.

## Results

### Lumican is enriched in human VAT and adipocytes from subjects with DM

**Figure 1.**
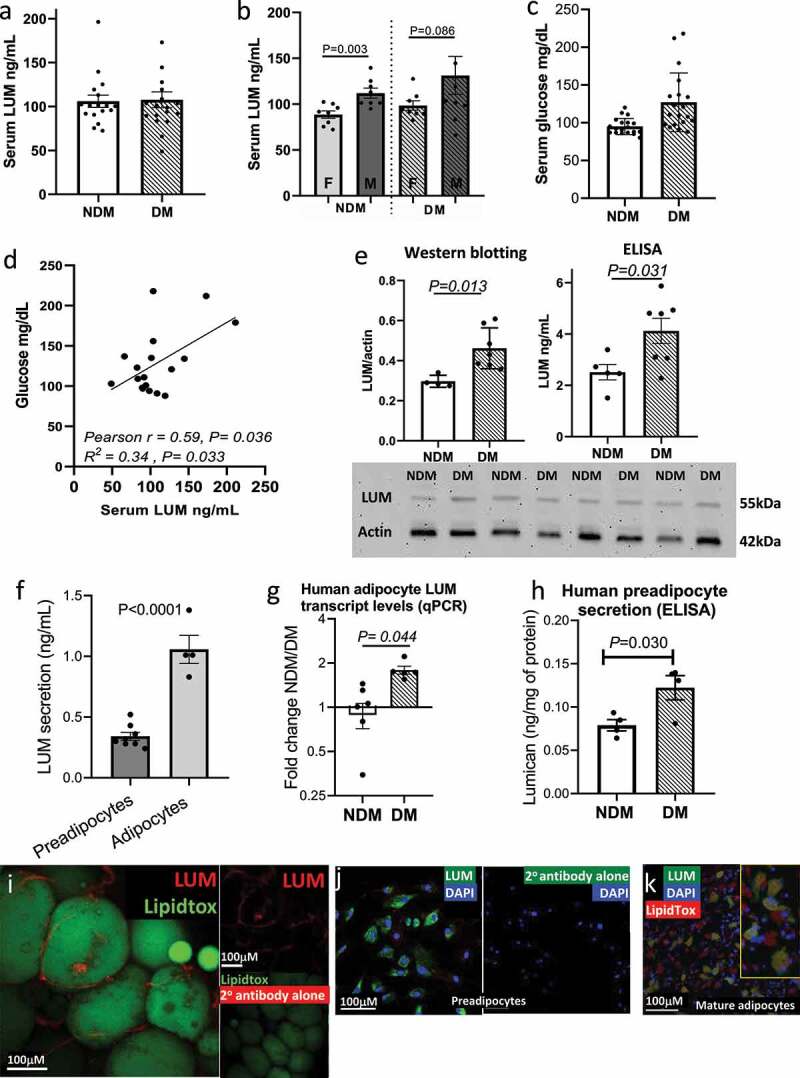
Lumican is enriched in VAT and adipocytes in human DM. (a,b) Concentrations of lumican (ng/mL) in serum from male (n = 16) and female (n = 14) patients with (DM) and without (NDM) type 2 diabetes (n = 19 DM, 19 NDM); data displayed in aggregate (a) and disaggregated by sex (b). (c) Serum glucose levels from same cohort. (d) Correlation analysis of fasting glucose and lumican concentrations in serum of patients with DM (n = 19). No correlation was observed in NDM subjects. (e) Lumican protein quantification in VAT lysates from DM (n = 5) and NDM (n = 7) subjects analysed by Western blot and ELISA. Actin expression was used as loading control for Western blotting. Lumican concentrations detected by ELISA were normalized by total protein in VAT lysates. (f) Lumican secretion in supernatants from human VAT preadipocytes (n = 8) and adipocytes (n = 4) cultured in vitro. Samples were collected at confluence and after adipogenic differentiation for 14 d, respectively, and analysed by ELISA. (g) qPCR analysis of *LUM* in *in vitro* differentiated VAT adipocytes. Data shown as fold change over NDM samples. (h) Lumican secretion in supernatants from human VAT preadipocytes from NDM (n = 4) and DM (n = 4) subjects cultured *in vitro*, demonstrating increased lumican secretion by DM preadipocytes. (i) Representative confocal microscopy image (n = 6 samples, 20X objective) of human VAT immuno-labelled with lumican (red) and LipidTox (green). Image lower right shows VAT stained with secondary antibody only as a control, confirming lack of autofluorescence and lack of non-specific staining. (j) Representative confocal microscopy image (n = 6 samples, 20X objective) of lumican (green) expression in VAT human preadipocytes. Nuclei are stained in blue. Image on right shows preadipocytes stained with secondary antibody only as a control, confirming lack of autofluorescence and lack of non-specific staining. (k) Representative immunofluorescence microscopy (n = 6 samples,10x objective) of mature VAT adipocytes differentiated for 14 d *in vitro* then immunostained with lumican (green). Lipid droplets are shown in red and nuclei in blue. Yellow rectangle shows zoomed field.

We evaluated lumican as a potential circulating biomarker in DM by measuring lumican concentrations in peripheral blood serum of DM and NDM subjects with obesity. Serum levels of lumican were similar between DM and NDM subjects (Figure 1(a)). When disaggregated by sex, serum lumican levels were higher in men compared to women, but increased levels of DM relative to NDM remained (Figure 1(b)). Serum glucose levels were, as expected, higher in DM relative to NDM subjects (Figure 1(c)). Linear regression analysis revealed a positive association between serum lumican concentrations and fasting blood glucose in DM, but not NDM subjects. No significant correlation was observed between serum lumican and age, BMI, HbA1c, triacylglycerol, total cholesterol, HDL, or LDL concentrations (Figure 1(d); Supplementary Table 2).

We next studied lumican expression in human VAT. We demonstrated increased lumican protein levels in DM relative to NDM intact VAT with Western blotting and ELISA for lumican detection (Figure 1(e)). To better define lumican expression at the cell level, we used qPCR to compare lumican secretion by preadipocytes and adipocytes *in vitro* and found that adipocytes secrete higher amounts of lumican compared with preadipocytes (Figure 1(f)). We next demonstrated increased *LUM* gene expression DM compared with NDM in *in vitro* cultured adipocytes using qPCR (Figure 1(g)). Finally, to confirm these results, we used ELISA to demonstrate increased lumican by preadipocytes from DM relative to NDM subjects in *in vitro* culture (Figure 1(h)).

To better localize lumican within adipocyte tissue, we used immunofluorescence microscopy of whole mount VAT biopsies from subjects with DM and demonstrated localization of lumican to the cell membrane of mature adipocytes and in the intercellular space between adipocytes, consistent with lumican expression by both preadipocytes and mature adipocytes in intact tissue (Figure 1(i)). Immunofluorescence microscopy of cultured cells revealed that lumican is expressed in some but not all VAT preadipocytes and adipocytes (Figure 1(j,k)).

Together, these results demonstrate that lumican expression is increased in VAT in DM and is expressed by subpopulations of both preadipocytes and mature adipocytes but to a greater degree by the latter.

### LUM regulates adipocyte metabolism

**Figure 2.**
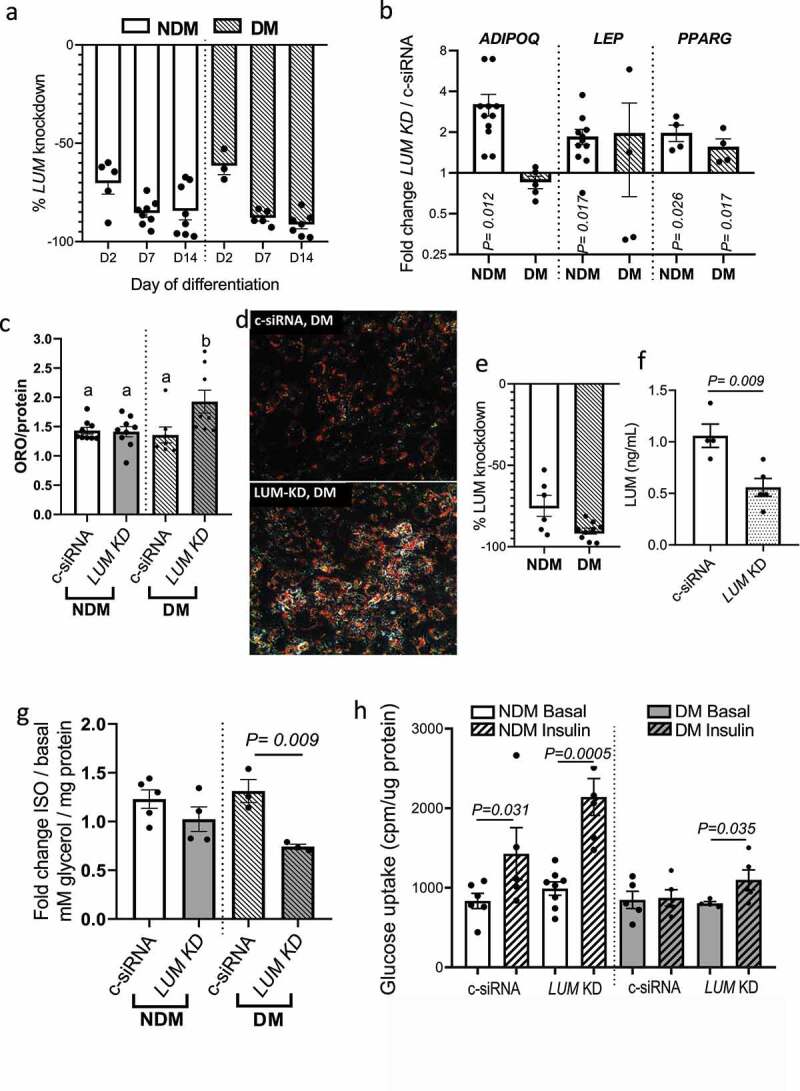
LUM knockdown rescues adipocyte cellular metabolism in DM human VAT. (a) LUM gene expression studied with qPCR in VAT adipocytes in response to siRNA LUM KD. Scrambled siRNA (c-siRNA) served as control. Results are shown as fold change over c-siRNA. (b) qPCR analysis of gene expression of *ADIPOQ, LEP and PPARG* in *LUM* KD in DM (n = 5) and NDM (n = 10) adipocytes at d 14 of differentiation in response to siRNA *LUM* KD. Data are shown as fold change over c-siRNA expression. (c) Effect of *LUM* KD on adipocyte adipogenic differentiation measured by Oil Red-O content in DM (n = 6) and NDM (n = 10) adipocytes. Data were calibrated by total protein concentration/well. (d) Representative images of increased lipid accumulation in DM *LUM KD* adipocytes. (e) Reduction in *LUM* gene expression and (f) LUM secretion of adipocytes transfected with LUM targeted siRNA on d 14 of differentiation. qPCR data shown as fold change expression over c-siRNA control. (g) Lipolytic responses in *LUM* KD and c-siRNA adipocytes from DM (n = 3) and NDM (n = 5) patients in response to stimulation with isoproterenol. Data shown as average fold change in glycerol concentrations released by stimulated adipocytes vs. non-stimulated adipocytes. P-value shown compares indicated bars. Not shown in figure are p-values comparing NDM vs. DM groups within each experimental arm (c-siRNA, LUM-KD); all such comparisons were not significant (NDM vs. DM groups within LUM-KD experimental arm p = 0.126). (h) Adipocyte glucose uptake ± insulin stimulation (200 nM, 40 min) in *LUM* KD and c-siRNA adipocytes from DM (n = 5) and NDM (n = 8) patients. Data is shown as cpm calibrated by total protein/well. P-values shown compare indicated bars. Not shown in figure are p-values comparing NDM vs. DM groups within each experimental arm (basal, insulin, c-siRNA, LUM-KD); all such comparisons were not significant, except for NDM vs. DM groups within the insulin (+)/LUM-KD experimental arm, for which p = 0.005.

To evaluate the role of lumican in regulating adipocyte function, we knocked down *LUM* in adipocytes using siRNA (LUM KD). We observed that *LUM* gene expression increased during proliferative expansion (prior to addition of differentiation media) in VAT preadipocytes *in vitro* (Supplementary Figure S2). Thus, we investigated the role of *LUM* on adipogenesis by knocking down *LUM* on d 0 and d 7 of adipogenic differentiation *in vitro*. Effectiveness of LUM KD was confirmed by qPCR at d 2 (D2), 7 (D7), and 14 (D14) of differentiation (Figure 2(a)). On D14, adipogenesis was assessed by qPCR and ORO staining. Relative to control cells transfected with scrambled siRNA (c-siRNA), LUM KD increased expression of *PPARG* in NDM and DM adipocytes and increased expression of *ADIPOQ* and *LEP* in NDM cells only (Figure 2(b)). Notably, LUM KD potentiated adipogenic lipid accumulation in adipocytes from DM VAT, but not NDM (Figure 2(c-d)). Overall, these results suggest that *LUM* may be involved in interfering in distinct phases of adipogenesis in a manner dependent on DM status.

Next, we studied the effect of LUM KD on adipocyte metabolism by knocking down LUM in differentiated adipocytes. qPCR analysis 48 h after transfection and lumican ELISA of cell culture supernatants were used to confirm *LUM* knockdown (Figure 2(e,f)). Adipocyte metabolism was assessed by lipolytic responses and insulin-stimulated glucose uptake. While there was no difference in isoproterenol-stimulated lipolysis between LUM KD and c-siRNA in NDM adipocytes, lipolysis was significantly decreased in LUM KD in DM adipocytes (Figure 2(g)). Importantly, LUM KD, compared with c-siRNA, rescued insulin sensitivity in DM adipocytes, and increased insulin response in NDM adipocytes, as demonstrated by insulin-stimulated glucose uptake (Figure 2(h)).

**Figure 3.**
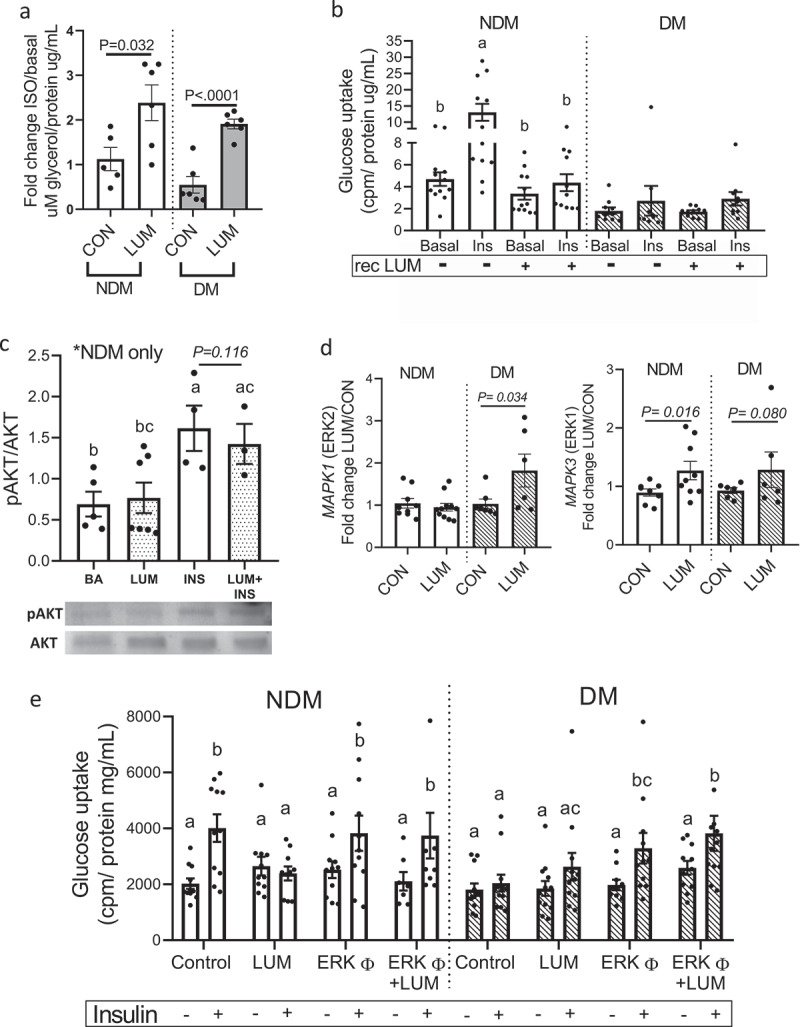
Increased lumican leads to adipocyte dysfunction through ERK signalling. In vitro differentiated VAT adipocytes were treated with recombinant human lumican or basal medium (CON) and then analysed for: (a) Lipolytic responses; data is shown as average fold change in glycerol concentrations released by stimulated adipocytes over basal concentrations from non-stimulated adipocytes. P-values shown compare indicated bars. Not shown in figure are p-values comparing NDM vs. DM groups within each experimental arm (CON, LUM); these comparisons were not significant (p = 0.098, 0.279 comparing NDM vs. DM groups within CON and LUM experimental arms respectively). (b) Glucose uptake in response to recombinant lumican. Different letters represent P ≤ 0.05 using Tukey multiple comparisons analysis. Not shown in figure are p-values comparing NDM vs. DM groups within each experimental arm (CON, LUM), for which p < 0.05 comparing NDM vs DM groups in all arms except the insulin(+)/rec Lum arm, for which p = 0.157. (c) pAKT phosphorylation in response to recombinant lumican. Different letters represent P ≤ 0.05 using Tukey multiple comparisons analysis. (d) Gene expression analysis of *LEP, MAPK1* (ERK2) and *MAPK3* (ERK1) assessed by qPCR. Data is shown as fold change expression over untreated, control (CON) cells. (e) Glucose uptake by adipocytes from DM (n = 7) and NDM (n = 6) patients treated for 24 h with ± lumican (LUM; 2 ug/mL) and ± 10 mM ERK inhibitor U0126 (ERKφ) in serum free medium. After 24 h, cells were treated or not with 200 nM insulin for 40 min. Data is shown cpm calibrated by total protein/well. Different letters represent P ≤ 0.05 using Tukey multiple comparisons analysis. Data for all graphs: mean ± SEM.

We next assessed the effects of human recombinant lumican on VAT adipocyte metabolism. The addition of recombinant lumican to adipocyte cultures increased the lipolytic responses in NDM and DM adipocytes (Figure 3(a)). Recombinant lumican impaired insulin-stimulated glucose uptake in NDM adipocytes but had no effect on glucose uptake in DM adipocytes, which are already insulin resistant (Figure 3(b)). To explore downstream mechanisms of lumican’s effects on IR, we studied pAKT signalling using phosphospecific Western blotting. We observed no effect of recombinant lumican on pAKT expression in NDM adipocytes (Figure 3(c)), suggesting that lumican may affect other up- or downstream molecules of insulin signalling cascade.

Together, these studies indicate that LUM exerts adverse effects on adipogenesis, lipolysis, and adipocyte insulin sensitivity and that LUM KD rescues the dysfunctional metabolic phenotype observed in DM adipocytes.

### Lumican impairs adipocyte metabolism via ERK signalling

Given the observed lack of effect of LUM on pAKT, along with previous reports linking the detrimental effects of lumican on cell migration and growth to ERK1/2 signalling [15,28], we next investigated whether ERK1/2 signalling mediates lumican effects on impairment of adipocyte insulin sensitivity. Recombinant lumican increased ERK2 gene expression in DM adipocytes and ERK1 expression in NDM adipocytes, with a trend towards increased ERK1 expression in DM cells (Figure 3(d)) but recombinant lumican did not affect the expression of a panel of ECM genes nor did lumican knockdown with siRNA: *CD44, COL1A1, COL1A2, COL6A1, H1F, ITGB1, MMP3, MMP11, MMP2* (Supplementary Figure S3). To test whether ERK1/2 mediates the detrimental effect of lumican on adipocyte insulin signalling, we treated adipocytes with an ERK inhibitor (U0126) [15] in the presence or absence of recombinant lumican, followed by glucose uptake assay. Inhibition of ERK1/2 abrogated lumican’s inhibitory effects on insulin-stimulated glucose uptake in NDM adipocytes and rescued insulin-stimulated glucose uptake in DM adipocytes (Figure 3(e)). These findings suggest that lumican regulates adipocyte insulin sensitivity via ERK1/2 pathway signalling.

## Discussion

Our results suggest a role for the proteoglycan lumican in adipocyte dysfunction in obesity-associated DM. We find that lumican expression is augmented in whole VAT and adipocytes in DM and exerts detrimental effects on adipocyte metabolic function. These observations are consistent with recent studies that implicate lumican in impairment of adipogenesis and insulin signalling. In *in vivo* murine models of obesity (ob/ob and db/db), AT *LUM* levels correlate with impaired systemic metabolism [29]. Treatment with lumican downregulated the expression of adipogenic transcription factors *C/EBPα* and *PPARγ* in mice 3T3-L1 cells while hyperglycemic/hyperinsulinemic conditions stimulated adipocytes to release lumican [9]. Similarly, in our study, we observed increased gene expression of lumican in insulin resistant DM adipocytes. Additionally, our data also demonstrate that *LUM* knockdown rescued DM adipocyte metabolism by improving insulin sensitivity, decreasing lipolysis, and increasing adipogenesis. These findings support an autocrine mechanism in which adipocyte-derived-lumican, elevated in DM, impairs VAT adipocyte metabolism.

Lumican is abundant in adipocyte ECM in 3T3-L1 cell cultures [30] and increases in expression and concentration intra- and extracellularly as adipogenesis progresses [9,31]. Similarly, we demonstrated increasing *LUM* expression during the proliferative phase of adipogenesis *in vitro*, copious lumican expression in the cytoplasm of *in vitro* cultivated human preadipocytes and adipocytes and secretion of lumican by adipocytes into the culture medium. The profuse expression of lumican in the periphery of adipocytes and intercellular space/ECM in human whole VAT mounts in our study suggests a potential role for this proteoglycan in regulating adipocyte-ECM function. The effects of lumican on ECM and fibrosis have been as previously reported in human and mouse models. Transgenic mice, constitutively expressing active HIF-1a in adipose tissue and fed a high-fat diet, developed adipose tissue fibrosis and exhibited an increased expression of *LUM* and other ECM genes [32]. *In vitro*, lumican-treated fibroblasts differentiated into fibrocytes under a pro-inflammatory stimulus with TNF-alpha [33], while human preadipocytes expressing a pro-inflammatory phenotype overexpressed *LUM* and other ECM genes such as fibronectin, collagens, and metalloproteinases (MMP) [34]. Notably, we did not observe the effect of human recombinant lumican on NDM and DM adipocyte expression of ECM and fibrosis-associated genes. Further research is needed to establish to what extent lumican is released under pathophysiological conditions to exert its effects on the ECM.

A knowledge gap exists regarding lumican signalling pathways, which are variable depending on cell type. Intracellular mechanisms of lumican action are reported only in the context of wound healing and cancer progression [28], and implicate integrins and activation of ERK1/2^34 15^. ERK1/2 are Mitogen-Activated Protein Kinases (MAPK) and their defective activation is associated with IR in skeletal muscle and adipose tissue [35,36], but lumican signalling in adipose tissue has not been studied. Our data demonstrate that lumican induces ERK1/2 expression, while ERK1/2 inhibition blocks lumican-mediated impairment of insulin sensitivity in NDM and DM adipocytes. These results imply the involvement of ERK 1/2 signalling pathway on lumican’s detrimental effects on human adipocyte metabolism and highlight the potential of lumican-ERK signalling as a target for manipulating adipose tissue metabolism.

*In vitro* cell culture models have limitations. We demonstrate cellular insulin resistance with a functional glucose uptake assay as well as reductions in Akt phosphorylation, supporting that lumican mediates its effects on adipocyte insulin resistance at least in part via an Akt-related mechanism, but our data cannot rule out other mechanisms of lumican-mediated insulin resistance in addition to Akt Ser473 phosphorylation, including regulation of other insulin signalling mediators or GLUT4 translocation. Future studies will be necessary to determine if lumican regulates ERK at the level of protein phosphorylation, in addition to gene expression as shown in this study. In these experiments, we used recombinant lumican produced in a bacterial expression system, which may not be glycosylated in the same manner as *in vivo*. Our data demonstrate that while *in vitro* responses to lumican were similar between sexes, serum lumican levels were lower in women compared to men. Further research will be necessary to define sex differences in lumican function at the tissue level. Finally, it is of interest that some effects of lumican were observed in NDM but not DM subjects. We hypothesize that elevated lumican levels in DM tissues, along with other DM-specific detrimental effects on adipocyte function, may lead to impaired metabolic function in DM adipocytes that may render these cells less susceptible to further impairment by exogenous lumican in *in vitro* culture.

Our findings implicate lumican as a potential mediator of cellular metabolic defects in DM, including impaired adipogenesis, increased intracellular lipid storage and lipolysis, and IR, and suggest that this understudied ECM molecule is a putative link mediating ECM regulation of adipocyte metabolism.

## Supplementary Material

Supplemental Material

## Data Availability

Data from this study are available from the corresponding author upon reasonable request.
