# Supplementary material for: Lumican modulates adipocyte function in obesity-associated type 2 diabetes
Source: Adipocyte. 2022 Dec 6;11(1):665–75. doi: 10.1080/21623945.2022.2154112 (PMC9728465; doi:10.1080/21623945.2022.2154112)
Supplement: Supplemental Material [file KADI_A_2154112_SM6130.zip › supplement/CSB Supplementary Figures REV.pptx]

## Slide 1
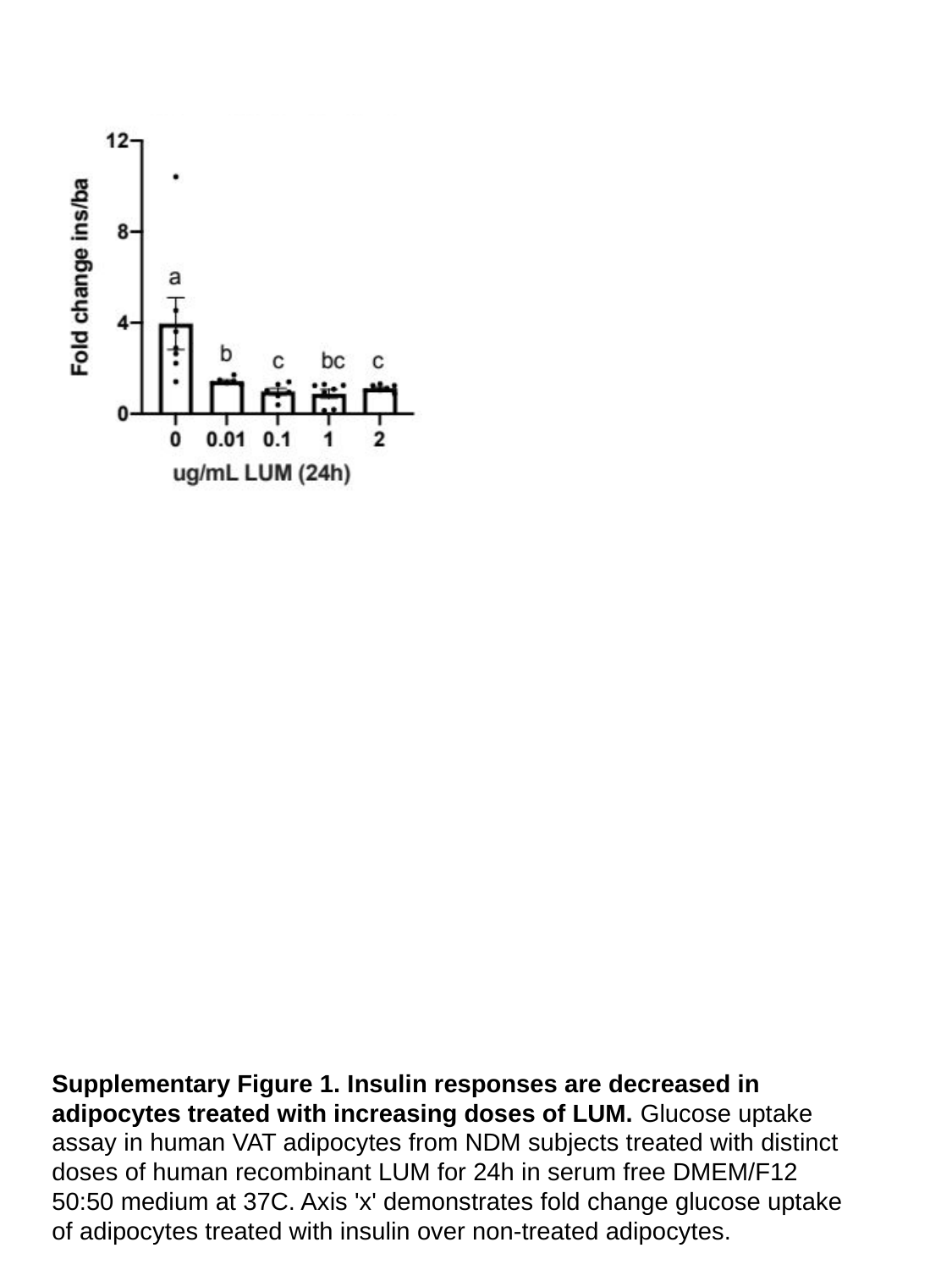

Supplementary Figure 1. Insulin responses are decreased in adipocytes treated with increasing doses of LUM. Glucose uptake assay in human VAT adipocytes from NDM subjects treated with distinct doses of human recombinant LUM for 24h in serum free DMEM/F12 50:50 medium at 37C. Axis 'x' demonstrates fold change glucose uptake of adipocytes treated with insulin over non-treated adipocytes.

## Slide 2
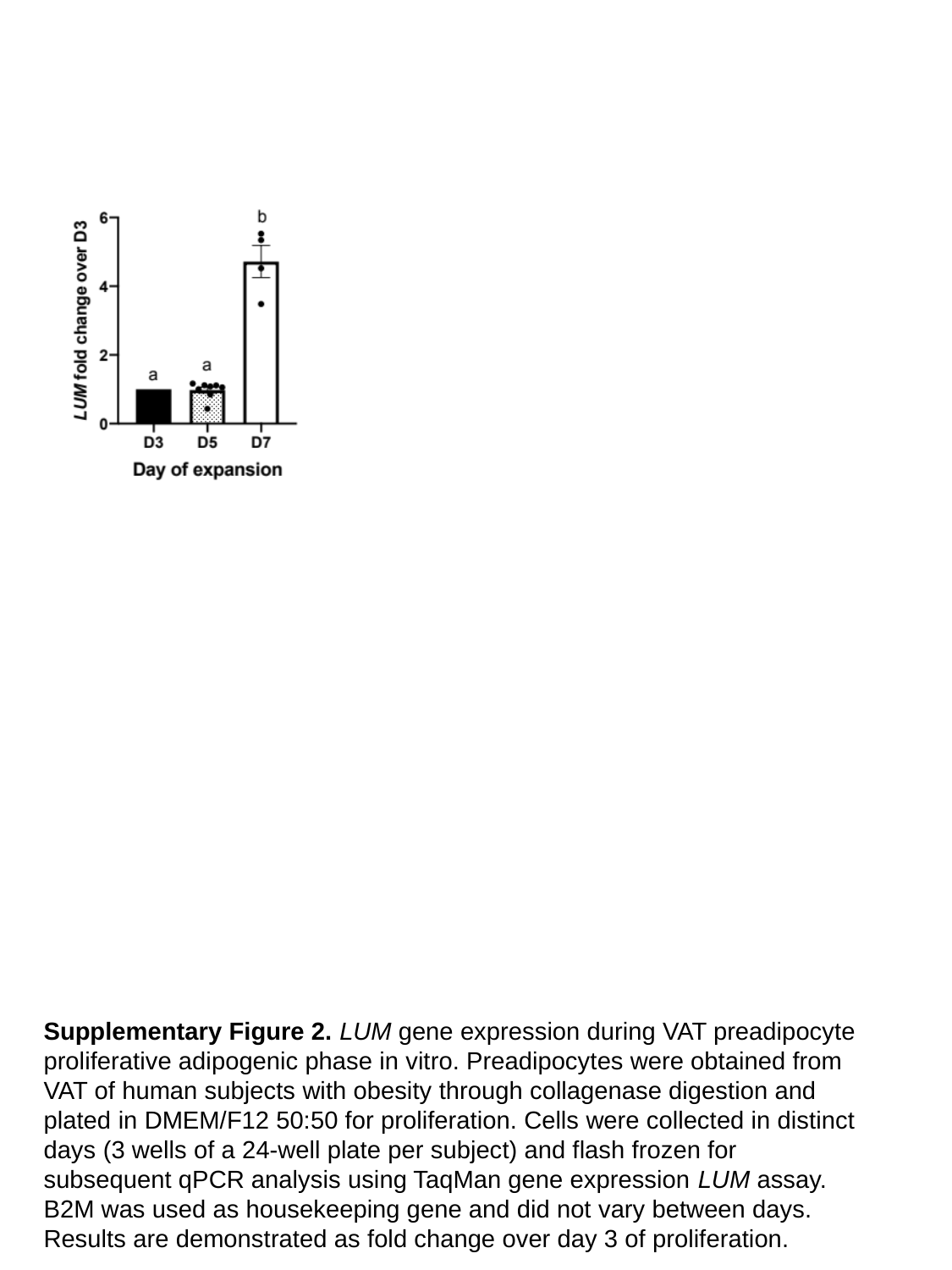

Supplementary Figure 2. LUM gene expression during VAT preadipocyte proliferative adipogenic phase in vitro. Preadipocytes were obtained from VAT of human subjects with obesity through collagenase digestion and plated in DMEM/F12 50:50 for proliferation. Cells were collected in distinct days (3 wells of a 24-well plate per subject) and flash frozen for subsequent qPCR analysis using TaqMan gene expression LUM assay. B2M was used as housekeeping gene and did not vary between days. Results are demonstrated as fold change over day 3 of proliferation.

## Slide 3
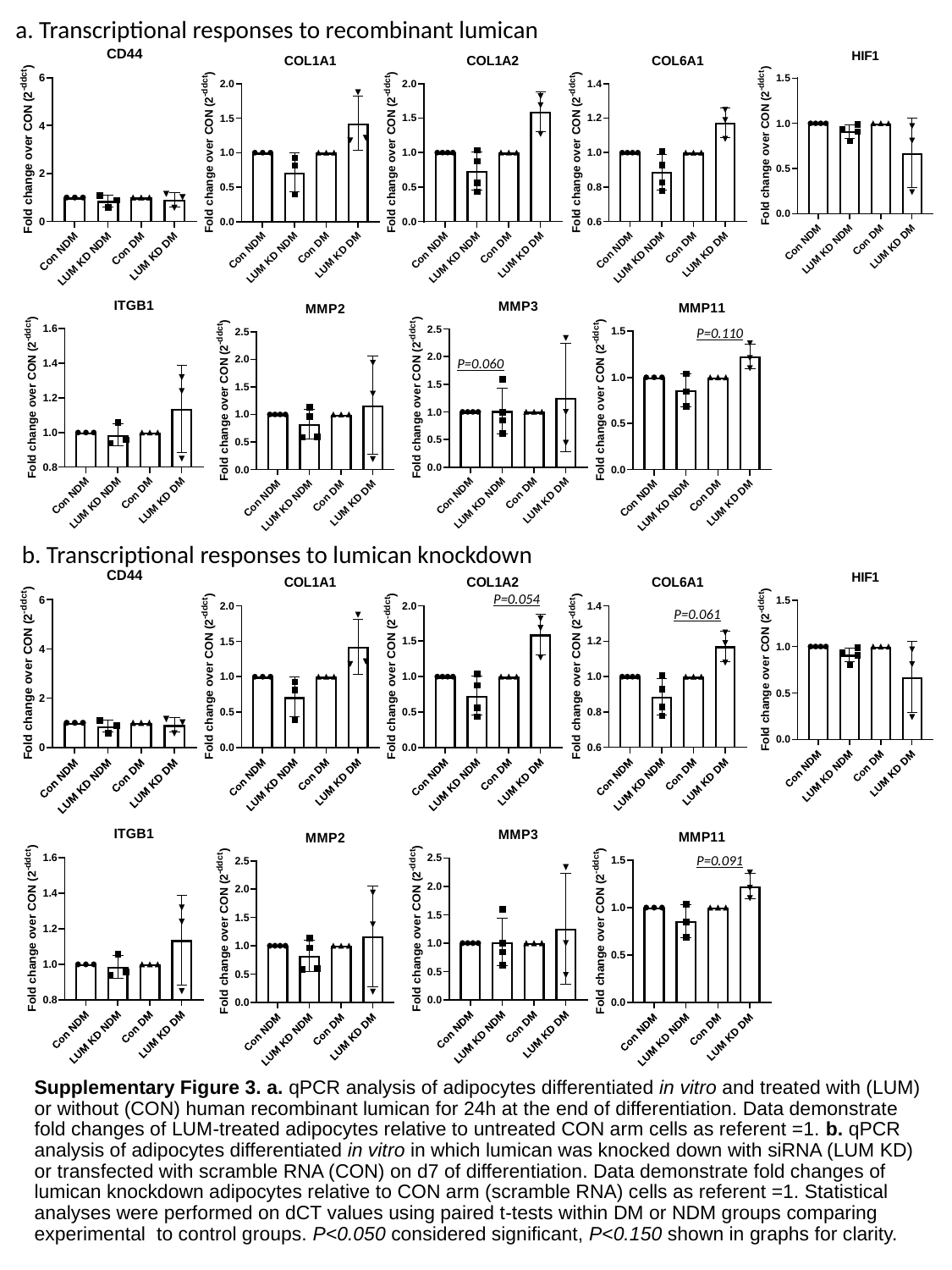

a. Transcriptional responses to recombinant lumican
P=0.110
P=0.060
b. Transcriptional responses to lumican knockdown
P=0.054
P=0.061
P=0.091
Supplementary Figure 3. a. qPCR analysis of adipocytes differentiated in vitro and treated with (LUM) or without (CON) human recombinant lumican for 24h at the end of differentiation. Data demonstrate fold changes of LUM-treated adipocytes relative to untreated CON arm cells as referent =1. b. qPCR analysis of adipocytes differentiated in vitro in which lumican was knocked down with siRNA (LUM KD) or transfected with scramble RNA (CON) on d7 of differentiation. Data demonstrate fold changes of lumican knockdown adipocytes relative to CON arm (scramble RNA) cells as referent =1. Statistical analyses were performed on dCT values using paired t-tests within DM or NDM groups comparing experimental to control groups. P<0.050 considered significant, P<0.150 shown in graphs for clarity.
