# Supplementary material for: Lumican modulates adipocyte function in obesity-associated type 2 diabetes
Source: Adipocyte. 2022 Dec 6;11(1):665–75. doi: 10.1080/21623945.2022.2154112 (PMC9728465; doi:10.1080/21623945.2022.2154112)
Supplement: Supplemental Material [file KADI_A_2154112_SM6130.zip › supplement/CSB Supplementary Tables.docx]

**Supplementary materials**

**Supplementary Table 1.** Demographic and clinical information of female obese patients with (DM) and without (NDM) type 2 diabetes included in this study.

|  | | **NDM (n=19)** | **DM (n=19)** | **P value*** |
| --- | --- | --- | --- | --- |
| Subjects’ demographics | Age (mean ± SD, years) | 41 ± 9 | 45 ± 9 | 0.130 |
|  | BMI (mean± SD, kg/m^2^) | 46 ± 6 | 46 ± 6 | 0.880 |
|  | Sex (% female) | 52.63 | 47.37 | >0.999 |
| Blood metabolic profile | HbA1c (%) | 5.37 ± 0.3 | 7.10 ± 0.9 | <0.001 |
|  | Glycemia (mg/dL) | 95 ± 11 | 127 ± 39 | 0.001 |
|  | TG | 136 ± 37 | 181 ± 103 | 0.179 |
|  | Cholesterol | 191 ± 56 | 173 ± 44 | 0.344 |
|  | HDL | 45 ± 12 | 46 ± 9 | 0.364 |
|  | LDL | 129 ± 53 | 91 ± 29 | 0.027 |
| Comorbid diseases (% of patients) | Sleep apnea | 70% | 60% | 0.660 |
|  | Hypertension | 32% | 79% | 0.008 |
|  | Hyperlipidemia | 21% | 40% | 0.301 |
| Medications (% of patients) | ACE inhibitor | 0% | 44% | 0.022 |
|  | Beta-blocker | 0% | 22% | 0.110 |
|  | Insulin | 0% | 56% | 0.006 |
|  | Metformin | 0% | 78% | <0.001 |
|  | Statin | 0% | 44% | 0.022 |
|  | Sulfonylurea | 0% | 22% | 0.110 |
|  | Thiazolidinedione | 0% | 0% | >0.999 |
|  | GLP-1 agonist | 0% | 22% | 0.110 |
|  | DPP-4 Inhibitor | 0% | 22% | 0.110 |

*P-values were assessed by two-tailed t-tests between NDM and DM.

**Supplementary Table 2.** Table 1. Pearson correlation analysis* of serum lumican concentrations with demographic characteristics and blood metabolites in human subjects with DM.

|  | **Age (years)** | **BMI** | **Hba1c (%)** | **Glycemia (mg/dL)** | **Total cholesterol (mg/dL)** | **TAG (mg/dL)** | **HDL (mg/dL)** | **LDL (mg/dL)** |
| --- | --- | --- | --- | --- | --- | --- | --- | --- |
| **Pearson r** | 0.116 | 0.068 | 0.024 | 0.369 | 0.088 | -0.262 | 0.083 | -0.186 |
| **95% confidence interval** | -0.2313 to 0.4369 | -0.2767 to 0.3969 | -0.3167 to 0.3593 | 0.03462 to 0.6283 | -0.3024 to 0.4527 | -0.5895 to 0.1395 | -0.3068 to 0.4488 | -0.5479 to 0.2355 |
| **R squared** | 0.013 | 0.005 | 0.001 | 0.136 | 0.008 | 0.069 | 0.007 | 0.034 |
| **P value (two-tailed)** | 0.514 | 0.703 | 0.892 | **0.032** | 0.663 | 0.196 | 0.681 | 0.385 |
| **Number of XY Pairs** | 34 | 34 | 34 | 34 | 27 | 26 | 27 | 24 |

LUM: Serum LUM in ng/mL; CHOL: Total serum cholesterol; TAG: serum triacylglycerols

*two-tailed analysis
